# Lung endothelial cell senescence impairs barrier function and promotes neutrophil adhesion and migration

**DOI:** 10.1007/s11357-025-01517-9

**Published:** 2025-01-16

**Authors:** Maliheh Najari Beidokhti, Nuria Villalba, Yonggang Ma, Amanda Reynolds, Juan Hernandez Villamil, Sarah Y. Yuan

**Affiliations:** 1 Department of Molecular Pharmacology and Physiology, University of South Florida, Morsani College of Medicine, 12901 Bruce B. Downs Blvd., Tampa, FL USA; 2 Department of Surgery, University of South Florida, Morsani College of Medicine, Tampa, FL USA

**Keywords:** Aging, Endothelial cells, Inflammation, Neutrophils, Permeability, Senescence

## Abstract

Cellular senescence contributes to inflammation and organ dysfunction during aging. While this process is generally characterized by irreversible cell cycle arrest, its morphological features and functional impacts vary in different cells from various organs. In this study, we examined the expression of multiple senescent markers in the lungs of young and aged humans and mice, as well as in mouse lung endothelial cells cultured with a senescence inducer, suberoylanilide hydroxamic acid (SAHA), or doxorubicin (DOXO). We detected increased levels of p21, γH2AX, and SA-β-Gal and decreased Ki-67 and Lamin B1 in aged lungs and senescent lung endothelial cells. Importantly, the expression of senescent markers was associated with an inflammatory response in aged mouse lungs characterized by neutrophil infiltration, increased expression of intercellular adhesion molecule 1 (ICAM-1), and decreased protein levels of VE-cadherin and ZO-1. As the latter two are critical constituents of endothelial cell–cell junctions, we hypothesized that their decreased expression could lead to compromised junction barrier integrity. Indeed, senescent endothelial cells (ECs) exhibited impaired barrier properties, as measured by increased permeability to solutes of small size (3-kD) and albumin (70-kD). When co-cultured with neutrophils, senescent ECs and their supernatant promoted neutrophil chemotaxis and trans-endothelial migration. Taken together, our results suggest that lung EC senescence weakens cell–cell junctions, impairs barrier function, and promotes neutrophil adhesion and migration, which may contribute to the development of inflammation and related pathologies in the lungs during aging.

**Supplementary Information:**

The online version contains supplementary material available at 10.1007/s11357-025-01517-9.

## Introduction

Cellular senescence is defined as the permanent exit from the cell cycle, leading to loss of proliferative capability. Senescence can be induced by a variety of intrinsic or extrinsic stress, including telomere attrition, oncogene activation, constant DNA damage, mechanical stress, mitochondrial dysfunction, nutrient imbalance, and infection [1, 2]. Morphologically, senescent cells become enlarged and flattened with an increase in the cytoplasm-to-nucleus ratio [3]. Senescent cells are resistant to apoptosis due to anti-apoptotic signaling activation [4]. Several cellular senescence markers have been recognized. Enhanced expression of p16 and p21, cyclin-dependent kinase inhibitors, causes cell cycle arrest and protects senescent cells from death [5]. Lamin B1 plays a crucial role in preserving structural integrity of the nucleus, and its expression decreases during senescence [6]. Senescent cells also exhibit increased levels of γH2AX, a marker of DNA damage and telomere shortening [7]. Because of defective lysosomal turnover or alterations in autophagy, senescence-associated-β-galactosidase (SA-β-Gal) activity is also increased during cellular senescence [8]. In addition, senescent cells secrete a multitude of pro-inflammatory cytokines, chemokines, growth factors, and enzymes, referred to as senescence-associated secretory phenotype (SASP). Senescent cells accumulate in various tissues and organs during aging [9] and contribute to age-related diseases. Clearance of senescent cells has been shown to attenuate tissue dysfunction, improve physical function, and extend healthy lifespan [10–12].

The lungs are constantly exposed to the air and aerosol pathogens or pollutants, rendering them prone to infection, inflammation, and other insults. While aging affects every organ, the lungs are described as the most susceptible organ for disease in elderly people [13], where cellular senescence facilitates age-associated pathologies. This is supported by the findings that removal of senescent cells improves lung function in aged mice [14].

The lungs are a highly vascularized organ composed largely of microvessels that conduct gas exchange between the alveolar space and circulating blood [15]. The wall of these microvessels is primarily composed of a monolayer of ECs that connect to each other via cell–cell junctions, namely adherens junctions (AJs) and tight junctions (TJs) [16], providing a semi-permeable barrier that controls the movement of blood cells, proteins, and fluids across the microvascular wall. Junction opening or reduced expression facilitates the paracellular transport of fluid, proteins, and immune cells [16]. In addition, the endothelium regulates immune cell trafficking, activation, and function through differential expression of adhesion molecules, including ICAM-1 [15]. Endothelial dysfunction typically leads to plasma leakage, leukocyte adhesion and diapedesis, and coagulopathy; these pathological responses are exacerbated in aging.

In this study, we investigated the hypothesis that during aging, lung EC senescence impairs barrier function and promotes neutrophil adhesion/migration. Our study is unique as it provides a comparative analysis of multiple markers across human lungs, mouse lungs, and cultured lung ECs. More importantly, we provide functional analyses of the junction barrier property and inflammatory response in senescent lung ECs, contributing to a better understanding of the molecular basis of aging-related pathologies in the lungs.

## Methods

### Human lung tissue samples

Human lung tissues were provided by the LifeLink Foundation, a Tampa-based nonprofit corporation operating federally certified organ procurement organizations across Florida, Georgia, and Puerto Rico. An MTA has been executed enabling transfer of non-transplantable human organs or tissues to the labs at the University of South Florida Morsani College of Medicine for translational research. While experiments in tissues from decedents do not meet the NIH definition of human subject research, donors were de-identified according to HIPAA/IRB standards, with their age, sex, medical history, diagnosis, and cause of death recorded. Organs were recovered by designated transplant surgeons, processed according to the standard transplant protocols, and transported via authorized medical carriers. Tissue samples were collected from lungs, preserved in cryogenic molds using Tissue Plus® O.C.T. compound (Fisher HealthCare, Waltham, MA; catalog no. 4585), snap frozen in liquid nitrogen, and stored at –80 °C for future experimentation. Tissue sections were cut to 1-μm thickness for immunofluorescence analysis and SA-β-Gal staining.

### Animal studies

All experimental animal protocols were approved by the Institutional Animal Care and Use Committee at the University of South Florida and were conducted in accordance with the Guide for Care and Use of Laboratory Animals. Male C57BL/6 J mice purchased from the Jackson Laboratory (Bar Harbor, ME) were used in this project at the ages of 3 (young) and 19 (old) months old.

### Perfusion and mouse tissue collection

Mice were anesthetized with urethane and cardially perfused with PBS, followed by 4% paraformaldehyde (PFA). The collected tissues were fixed in 4% PFA for 24 h, washed with PBS, and transferred to 70% ethanol until tissue processing. The tissue was then embedded in paraffin and sectioned (4 μm thick) using a microtome. For “fresh-frozen” preparations, animals were cardially perfused with PBS. Then, the lungs were dissected, embedded in Tissue Plus® O.C.T. compound, snap frozen in liquid nitrogen, and sectioned (15 μm thick) using a cryostat (Leica CM1950).

### Immunofluorescence of senescent markers in lung tissue

Paraffin-embedded mouse lung sections were deparaffinized and subjected to appropriate antigen retrieval at 95 °C for 20 min. After permeabilization and blocking, the slides were incubated with primary antibodies p21 (1:50; Santa Cruz Biotechnology, Dallas, TX; catalog no. sc-6246), Ki-67 (1:20; Abcam, Waltham, MA; catalog no. ab16667), Lamin B1 (1:100; Abcam; catalog no. ab16048), or γH2AX (1:50; Abcam; catalog no. ab81299) overnight at 4 °C, washed, and then incubated with secondary antibodies for 1 h at room temperature. Slides were incubated with DAPI (1:2000; Thermo Scientific, Waltham, MA; catalog no. 62248) for 20 min at room temperature, and cover slipped using ProLong™ diamond antifade mountant (Invitrogen, Waltham, MA; catalog no. P36962). Images were captured using a Leica SP8 confocal microscope (Leica Microsystems, Weltzar, Germany).

Snap-frozen human and mouse lung sections were fixed in ice-cold methanol or 4% PFA for 15 min, washed with PBS, permeabilized, blocked, and incubated with primary antibodies: p21 (1:50; Abcam; catalog no.109520 or 1:50; Santa Cruz Biotechnology for human and mouse, respectively), ZO-1 (1:100; Invitrogen; catalog no. 40–2200), VE-cadherin (1:50; Cell Signaling Technology; catalog no. D87F2 or 1:100, BD Pharmingen; catalog no. 555289, for human and mouse, respectively), Ki-67, Lamin B1, or γH2AX overnight at 4 °C. Slides were then washed and incubated with secondary Alexa Fluor® 488 antibodies (1:500; Invitrogen; catalog no. A21206; A21202 and A21208) for 1 h at room temperature. Slides were rinsed, stained with DAPI, cover slipped, and imaged using a confocal microscope. Antibody dilutions were chosen based on previous series of titration experiments showing the best staining with minimum background fluorescence.

### SA-β-Gal staining in lung tissue

SA-β-Gal staining in lung tissue samples was performed as previously described [17]. Briefly, the sample slides with frozen tissue were washed twice with cold PBS, fixed with 2% formaldehyde + 0.2% glutaraldehyde in PBS on ice, and rinsed shortly in cold ultrapure water. The slides were incubated overnight at 37 °C in a non-CO_2_ incubator with fresh SA-β-Gal staining solution containing X-gal (20 mg/mL, pH 6.0; ThermoFisher; catalog no. 10113253) in dimethylformamide (1 mg/mL; MilliporeSigma; catalog no. 270547), citric acid/sodium phosphate buffer (40 mM, pH 6.0; Sigma-Aldrich; catalog no. C1909 and Fisher Scientific; catalog no. S375-500, respectively), potassium ferrocyanide (5 mM; Acros Organics; catalog no. 211095000), potassium ferricyanide (5 mM; Electron Microscopy Sciences; catalog no. 20150), sodium chloride (150 mM; Fisher Scientific; catalog no. S271-3), and magnesium chloride (2 mM; Fisher Scientific; catalog no. M35-500). Upon successful staining (deep blue color development in the tissue), slides were washed with PBS and cover slipped using mounting medium. Imaging was performed using an Olympus VS120 slide scanner.

### Endothelial cell culture and senescence induction in vitro

Mouse primary lung microvascular ECs (Cell Biologics; catalog no. C57-6011) were cultured in recommended medium (Cell Biologics; catalog no. M1168) and used at passages 5–7. In vitro senescence induction was carried out using two senescent inducers: SAHA and doxorubicin [17]. In brief, ECs were treated with SAHA (4 μM; Tocris; catalog no. 4652) for 8 days (drug was refreshed daily) and control (non-senescent) cells were cultured in parallel and treated with identical volumes of vehicle DMSO. Another group of ECs received doxorubicin (250 nM; Tocris; catalog no. 2252), and their control cells were treated with equal volumes of vehicle (sterile water) for 24 h, followed by a washout period for 7 days.

### Double staining for SA-β-Gal and EdU

Co-staining of SA-β-Gal and EdU was performed in cultured ECs as previously described [17]. Briefly, mouse primary lung microvascular ECs plated on 4-well cell culture chamber slides (Nest Scientific; catalog no. 230104) were treated with two senescent inducers (SAHA and doxorubicin) as mentioned above. The cells were then cultured in medium containing EdU (10 μM; Lumiprobe; catalog no. 10540) for 24 h. After washing with PBS, cells were fixed in 2% formaldehyde + 0.2% glutaraldehyde in PBS at room temperature, rinsed with PBS, and incubated in a non-CO_2_ incubator at 37 °C for 16 h with fresh SA-β-Gal staining solution, as described above. Upon successful staining, cells were then rinsed in PBS. After fixation and permeabilization, the cells were stained for 30 min in a dark at room temperature with fresh EdU staining solution containing CuSO4 (2 mM; Sigma-Aldrich; catalog no. 209198), sulfo-Cy3-azide (4 μM; Lumiprobe; catalog no. D1330), and sodium ascorbate (20 mg/mL; Sigma-Aldrich; catalog no. A4034) in PBS, washed with PBS, incubated with DAPI, and cover slipped using mounting medium. A Leica SP8 confocal microscope was used for imaging.

### Immunocytochemistry of senescent markers

After senescence induction, mouse lung ECs were fixed, permeabilized, blocked, and incubated with primary antibodies p21 (1:50; Abcam; catalog no. ab188224), Ki-67 (1:20; ThermoFisher Scientific; catalog no. MA5-14,520), Lamin B1, ZO-1, claudin-5 (1:50; Invitrogen; catalog no. 35–2500), or VE-cadherin (1:1000; Abcam; catalog no. ab205336) overnight at 4 °C. After washing, the slides were incubated with secondary Alexa Fluor® 488 antibodies (1:500; Invitrogen; catalog no. A21206; A21202) for 1 h at room temperature, followed by DAPI staining for 20 min at room temperature. Slides were then mounted and imaged using confocal microscopy. F-actin staining was performed using Alexa Fluor® 568 Phalloidin (1:40; Invitrogen; catalog no. A12380).

### Immunoblotting

Mouse lung tissue or lung microvascular ECs were lysed in 1 × RIPA lysis buffer (Millipore; catalog no. 20–188) containing protease and phosphatase inhibitors (Roche; catalog no. 11697498001; 04906845001). After centrifugation, the supernatant was collected, and protein quantitation was performed by Pierce BCA protein assay (ThermoFisher Scientific Inc.; catalog no. 23227). The lysates were then loaded onto 4–20% Tris–glycine gel (Bio-Rad; catalog no. 456–1094) and transferred onto a nitrocellulose membrane using Trans-Blot Turbo equipment. The membranes were stained with Revert™ 700 Total Protein Stain Kit (LI-COR Biosciences; catalog no. 926–11,016) and blocked in Intercept® (PBS) blocking buffer (LI-COR Biosciences; catalog no. 927–70,001) for 1 h at room temperature, probed with primary antibodies against p21 (1:1000), Lamin B1 (1:1000), ZO-1 (1:500), claudin-5 (1:250), ICAM-1 (1:250; Invitrogen; catalog no. MA5407), VE-cadherin (1:1000; Abcam; catalog no. ab205336), and β-actin (1:1000; LI-COR Biosciences; catalog no. 926–42,212; 926–42,210) overnight at 4 °C. After washing, membranes were probed with appropriate LI-COR secondary antibodies diluted in blocking buffer for 1 h at room temperature. The membranes were imaged using LI-COR Odyssey CLx system. The signal intensity of all protein bands was obtained by densitometry analysis and normalized to the total protein per lane (please see full-length Western blotting images; Supplemental Fig. S1-S7).

### Mouse bone marrow neutrophil isolation

Mouse bone marrow neutrophils were isolated as described previously [18, 19]. In brief, femurs and tibias were cut at one end, placed into a 0.5-mL tube, nested into a 1.5-mL Eppendorf tube, and centrifuged at 10,000 g for 30 s [20]. The bone marrow cells were collected, and red blood cells were lysed with 20 mL of 0.2% NaCl for 20–30 s, followed by addition of 20 mL of 1.6% NaCl. The cell pellet was then filtered with a cell strainer, counted using a Luna cell counter, and resuspended in 1 mL of PBS. Afterwards, 3 mL of Histopaque 1119 was gently added in a 15-mL conical tube, followed by 3 mL of Histopaque 1077, and then cell suspension (1 mL) was added on top of Histopaque 1077. After centrifugation at 2000 rpm for 30 min at 25 °C without brake, neutrophils were collected.

### Neutrophil adhesion assay

EC senescence was induced as mentioned above. Isolated bone marrow neutrophils (1 × 10^5^) were applied on senescent ECs, incubated for 2 h at 37 °C, and rinsed with PBS to remove all non-adherent neutrophils. Slides were then fixed in 4% PFA, rinsed with PBS and imaged using a bright-field microscopy. Slides were then permeabilized, blocked, and labelled with primary antibodies p21 (1:50; Abcam) and MPO (1:40; R&D systems; catalog no. AF3667) overnight at 4 °C. For paraffin-embedded mouse lung tissues, incubation with a primary antibody specific for neutrophils (1:100; Cederlane; catalog no. CL8993AP, clone 7/4) was performed. Cells or tissues were then incubated with secondary Alexa Fluor® 488 antibody (1:500; Invitrogen; catalog no. A21206; A11055) and Alexa Fluor® 568 antibodies (1:500; Invitrogen; catalog no. A10042; A11057 and A78946) for 1 h at room temperature. After washing, slides were stained with DAPI and imaged using a Leica SP8 scanning confocal microscope.

### Neutrophil chemotaxis assay

SAHA or doxorubicin-treated cells were seeded in the bottom chamber of gelatin-coated 24-well transwell plates (Costar, Corning, NY; catalog no. 3472) for 24 h. The following day, neutrophils (1 × 10^5^) were added to the top chamber insert (3 μm pore size) and incubated for 2 h at 37 °C with or without LTB4 (100 nM; Tocris; catalog no. 2307) as the chemoattractant agent in the bottom chamber. The number of neutrophils that chemotactically migrated into the bottom chamber was counted using a hemocytometer. The experiment was performed using supernatant of senescent cells in the bottom chamber as well.

### Neutrophil trans-endothelial migration assay

The assay was conducted according to our previously reported method with minor modifications [21]. Briefly, neutrophil trans-endothelial migration experiments were performed utilizing senescent and non-senescent ECs seeded on gelatin-coated 24-well transwell inserts of 3-μm pore size (Costar; catalog no. 3472) for 24 h. On the day of the experiment, neutrophils (1 × 10^5^) were added to the top chamber, incubated for 2 h at 37 °C with or without LTB4 in the bottom chamber as the chemoattractant. A hemocytometer was used to count the number of neutrophils transmigrated into the bottom chamber.

### Transwell permeability assay

To evaluate endothelial permeability, we utilized our previous described protocol with minor changes [22]. In brief, mouse primary lung microvascular ECs treated with two senescent inducers (SAHA and doxorubicin) were seeded (3 × 10^5^) on 0.33 cm^2^ inserts with pore size of 0.4 μm (Costar; catalog no. 3470) for 24 h. On the day of the experiment, 10 mg/mL of fluorescein isothiocyanate (FITC)-bovine serum albumin (FITC-BSA; Sigma-Aldrich; catalog no. A-9771) and 1 mg/mL rhodamine 3-kD dextran (Invitrogen; catalog no. D3307) were added to the top chamber. The passage of the fluorescence tracers was measured by collecting media from the bottom chamber, using a fluorescence microplate reader (SpectraMax M3; Molecular Devices) and apparent permeability coefficient (*P*_app_) of mouse primary lung microvascular EC monolayers were calculated as previously described [22].

### Statistical analysis

All data are presented as mean ± standard error mean (SEM). The comparison of young and old groups was analyzed by unpaired Student’s *t*-test. The comparison of SAHA or doxorubicin-treated cells and non-senescent cells was analyzed by paired Student’s *t*-test. Multiple comparisons were performed by one-way ANOVA with Tukey’s multiple comparison tests. Statistical analyses and graphs were performed with GraphPad Prism version 9 software. A value of *P* < 0.05 was considered statistically significant.

## Results

### Aged human lungs exhibit senescent characteristics and impaired endothelial cell–cell junctions

**Fig. 1:**
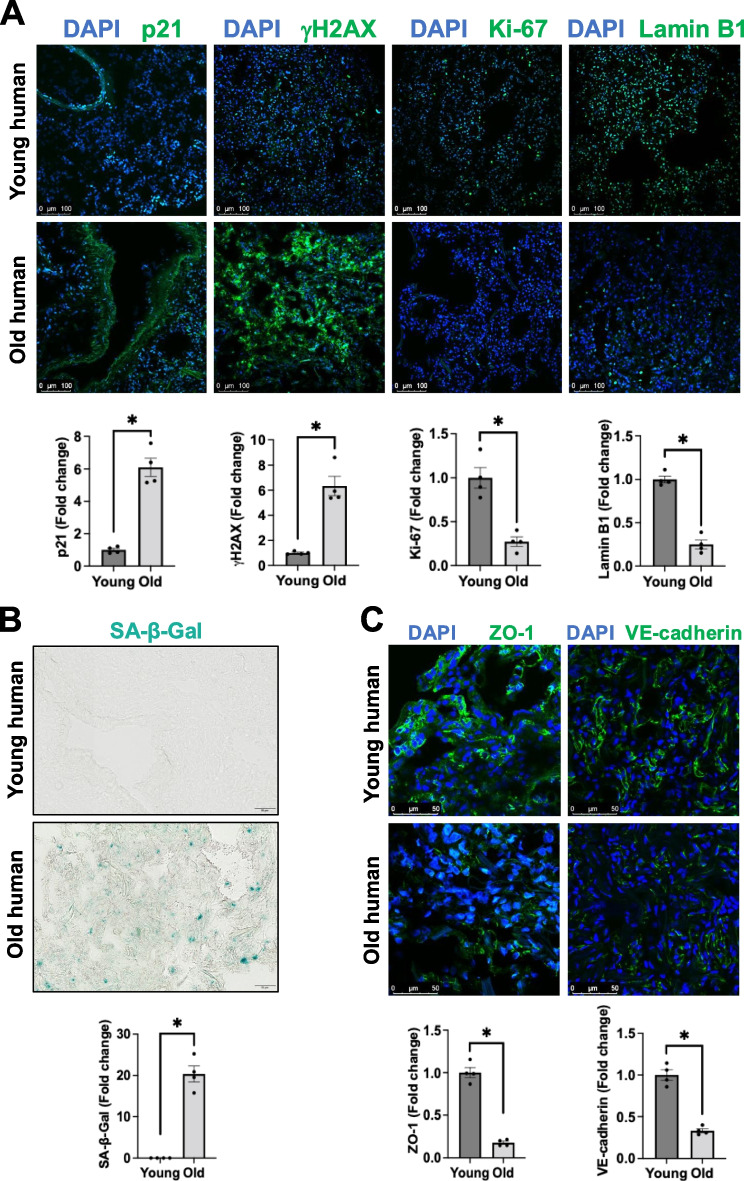
Aged human lungs display the senescent characteristics and impaired endothelial cell–cell junctions. **A** Representative confocal microscopy and image quantification revealed that aged human lungs exhibited increased expression of p21 and γH2AX and reduced expression of Ki-67 and Lamin B1. Scale bar = 100 μm.** B** Higher senescence associated β-galactosidase activity (SA-β-Gal, blue color) in aged than young human lungs. Scale bar = 50 μm.** C** Representative confocal microscopy and image quantification demonstrated that aged human lungs displayed lower levels of cell–cell junction molecules ZO-1 and VE-cadherin. Scale bar = 50 μm. Data are expressed as mean ± SEM and analyzed by unpaired Student’s *t*-test; *n* = 4 human lungs per group; *P < 0.05

First, we studied the expression of cellular senescence biomarkers in human lung specimens obtained from young (15–27 years old) and elderly (66–77 years old) donors without diagnosed lung diseases. Our immunofluorescence results revealed that lungs obtained from elderly showed higher protein levels of p21 and γH2AX and the loss of Ki-67 and Lamin B1 (Fig. 1A), indicative of a senescent phenotype. β-Galactosidase is a lysosomal hydrolase that breaks down β-galactosides into monosaccharides, and its levels are significantly increased in senescent cells [23]. For this reason, we measured SA-β-Gal activity. Our data demonstrated that SA-β-Gal activity (blue staining) was dramatically higher in elderly lung tissue than that of young lungs (Fig. 1B). These observations led us to validate markers of cell senescence in human lung tissue and their association with chronological aging.

An important function of ECs is to maintain barrier integrity and prevent vessel leakage at the steady state. To investigate whether EC senescence affects barrier structure, we measured the expression of two major junction proteins, ZO-1 and VE-cadherin, in young and aged human lungs with immunofluorescence. As shown in Fig. 1C, there was a significant loss of both ZO-1 and VE-cadherin staining in elderly samples compared to young tissue, implying that old human lungs show impaired integrity of endothelial cell–cell junctions.

### Aged mouse lungs display increased senescent cells and impaired EC junctions

**Fig. 2:**
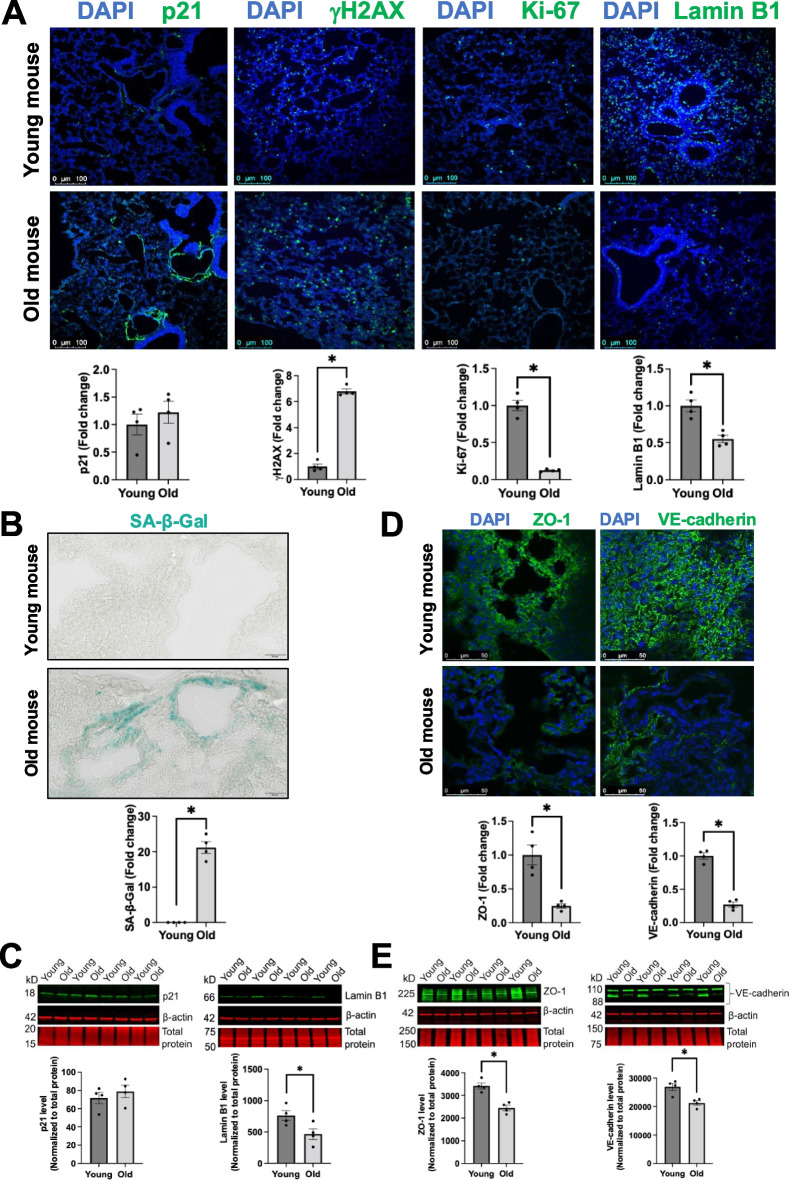
Aged mouse lungs exhibit the features of senescent cells and disrupted endothelial cell–cell junctions. **A** Representative confocal microscopy and image quantification revealed that aged mouse lungs exhibited increased expression of p21 and γH2AX and reduced expression of Ki-67 and Lamin B1. Scale bar = 100 μm.** B** Higher senescence associated β-galactosidase activity (SA-β-Gal, blue color) in aged than young mouse lungs. Scale bar = 50 μm.** C** Western blotting validated increased p21 expression and reduced Lamin B1 in aged mouse lungs. Full-length Western blotting images were provided in Supplemental Fig. S1. **D** Representative confocal microscopy and image quantification demonstrated that aged mouse lungs displayed lower levels of cell–cell junction molecules ZO-1 and VE-cadherin. Scale bar = 50 μm. Data are expressed as mean ± SEM and analyzed by unpaired Student’s *t*-test; *n* = 4 mice per group; *P < 0.05. **E** Western blotting validated decreased levels of ZO-1 and VE-cadherin in old mouse lungs. Data are expressed as mean ± SEM and analyzed by unpaired Student’s *t*-test; *n* = 4 mice per group; *P < 0.05. Full-length Western blotting images were provided in Supplemental Fig. S2

Next, we wanted to confirm human findings in mouse lung tissue. We compared the expression levels of senescence markers and endothelial junction proteins in the lungs from young (3 months old) and aged (19 months old) mice using both immunofluorescence and Western blot. Consistent with aged human lungs, aged mouse lungs exhibited upregulation of p21, γH2AX, and SA-β-Gal and downregulation of Ki-67 and Lamin B1 (Fig. 2A–C). Similarly, the lungs from aged mice have reduced expression of ZO-1 and VE-cadherin proteins compared to young controls (Fig. 2D, E). Collectively, our data demonstrate that aged lungs from humans and mice exhibit a phenotype of cellular senescence and endothelial junction impairment.

### Neutrophil recruitment increases in aged mouse lungs

**Fig. 3:**
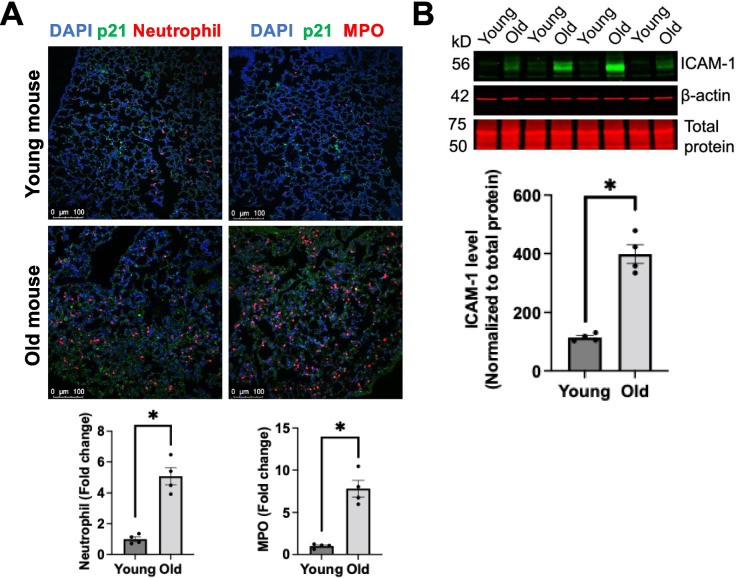
Neutrophil infiltration and ICAM-1 expression are increased in aged mouse lungs. **A** Representative confocal microscopy and image quantification showed more neutrophils in aged mouse lungs than young ones. Scale bar = 100 μm. Data are expressed as mean ± SEM and analyzed by unpaired Student’s *t*-test; *n* = 4 mice per group; *P < 0.05. **B** Western blotting revealed increased ICAM-1 levels in aged mouse lungs. Quantitative analysis of ICAM-1 (bottom panels). Values are band intensities normalized to total protein. Data are expressed as mean ± SEM and analyzed by unpaired Student’s *t*-test; *n* = 4 mice per group; *P < 0.05. Full-length Western blotting images were provided in Supplemental Fig. S3

The impairment of endothelial junction integrity causes microvascular hyperpermeability and may promote neutrophil extravasation [16]. Therefore, we hypothesized that aged lungs have more neutrophil infiltration. Indeed, our immunostaining with two neutrophil markers confirmed more neutrophil infiltration in the lungs of aged mice compared to young controls (Fig. 3A). Consistently, aged mouse lungs showed higher p21 levels compared to controls (Fig. 3A). ICAM-1 is known to mediate neutrophil adhesion to the endothelium and extravasation [24, 25]. Our Western blotting analysis showed that ICAM-1 protein expression was significantly elevated in aged mouse lungs compared to young counterparts (Fig. 3B). Together, these data indicate that senescent ECs may promote neutrophil infiltration into the lungs during aging possibly by upregulating ICAM-1.

### EC senescence induced by SAHA and doxorubicin

**Fig. 4:**
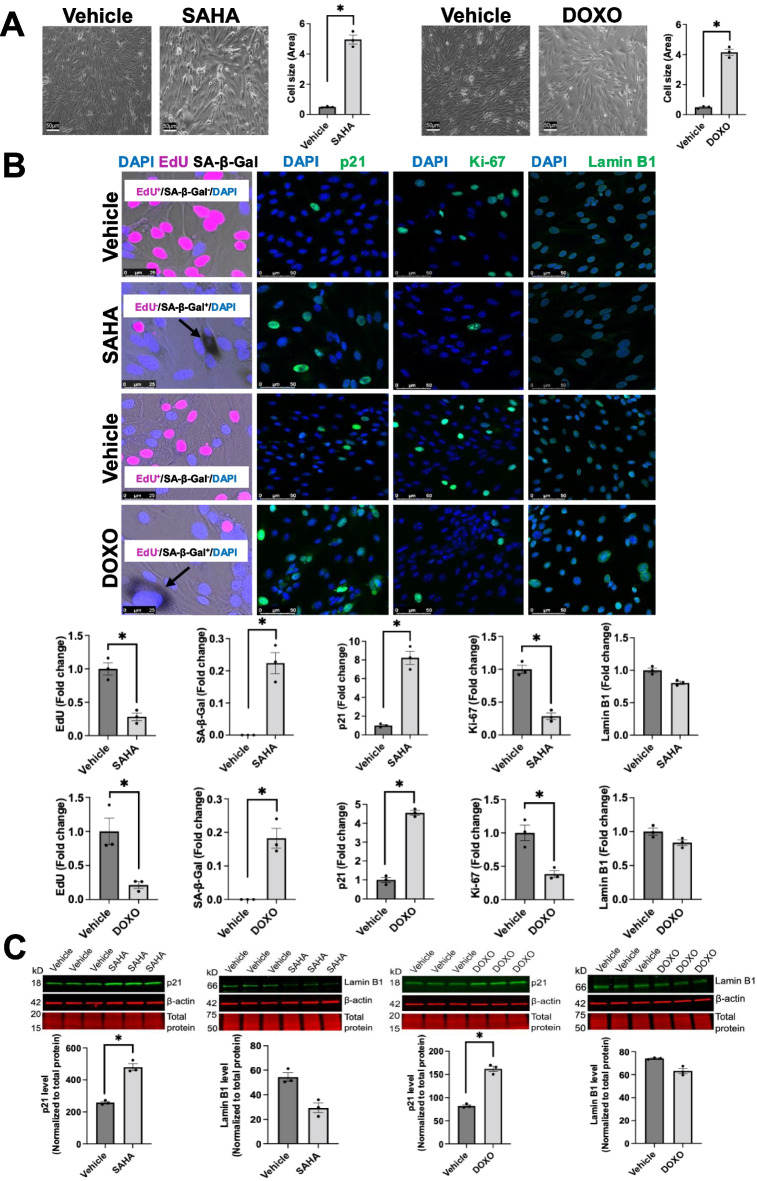
SAHA and doxorubicin induce cellular senescence in mouse lung microvascular ECs. **A** Representative bright-field microscopy and cell size quantification displayed enlarged morphology and irregular nuclear shape in SAHA- or doxorubicin (DOXO)-treated ECs compared to vehicle group (non-senescent cells). Scale bar = 50 μm. Data are expressed as mean ± SEM and analyzed by paired Student’s *t*-test; *n* = 3 replicates per group; *P < 0.05. **B** Representative confocal microscopy and image quantification showed increased expression of p21 and SA-β-Gal and decreased levels of EdU, Ki-67 and Lamin B1 in SAHA- or DOXO-treated ECs. Nucleus was counterstained with DAPI (blue). Scale bars = 25 μm and 50 μm. Data are expressed as mean ± SEM and analyzed by paired Student’s *t*-test; *n* = 3 replicates per group; *P < 0.05. **C** Western blotting revealed p21 upregulation and Lamin B1 downregulation as well as in old mouse lungs. Data are expressed as mean ± SEM and analyzed by paired Student’s *t*-test; *n* = 3 replicates per group; *P < 0.05. Full-length Western blotting images are provided in Supplemental Fig. S4

We induced senescence in mouse lung microvascular ECs by treatment with SAHA (4 μM) for 8 days or doxorubicin (DOXO; 250 nM) for 24 h with 7 days washout. SAHA and DOXO-treated lung ECs displayed typical morphological features of senescence, including enlarged morphology and irregular nuclear shape (Fig. 4A). Senescent ECs showed SA-β-Gal positivity and reduced staining of proliferation marker EdU. In addition, senescent ECs demonstrated enhanced expression of p21 and decreased expression of Ki-67 and Lamin B1 (Fig. 4B, C). These data confirm that SAHA and doxorubicin induce EC senescence.

### Senescent EC show increased permeability to different-sized molecules

**Fig. 5:**
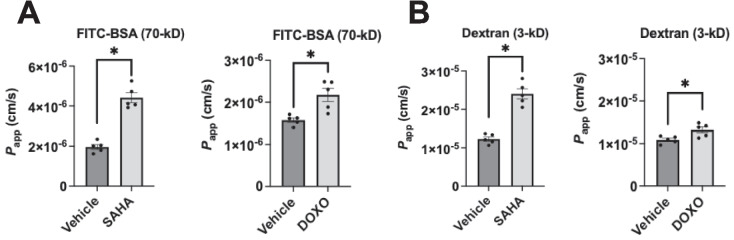
Senescent ECs show increased permeability to solutes of different sizes. **A** Endothelial permeability to FITC-BSA (70-kD) in SAHA- or DOXO-treated ECs is higher than that of vehicle-treated cells. **B** Endothelial permeability to dextran (3-kD) in SAHA- or DOXO-treated ECs is higher than that of vehicle-treated cells. Data are expressed as mean ± SEM and analyzed by paired Student’s *t*-test; *n* = 5 replicates per group; *P < 0.05

Our above data showed that aged human and mouse lungs show impaired barrier junctions. To determine whether this could cause increased permeability, we evaluated endothelial permeability by measuring *P*_app_. We assessed the permeability of a high and a low molecular weight tracer, FITC-BSA (~ 70 kD) (Fig. 5A) and rhodamine 3-kD dextran (Fig. 5B), respectively, using the transwell system. Our results revealed that senescent lung EC monolayers showed a remarkable increase in permeability to both small and large molecules compared to non-senescent ECs.

### Senescent ECs promote cell–cell junction disruption

**Fig. 6:**
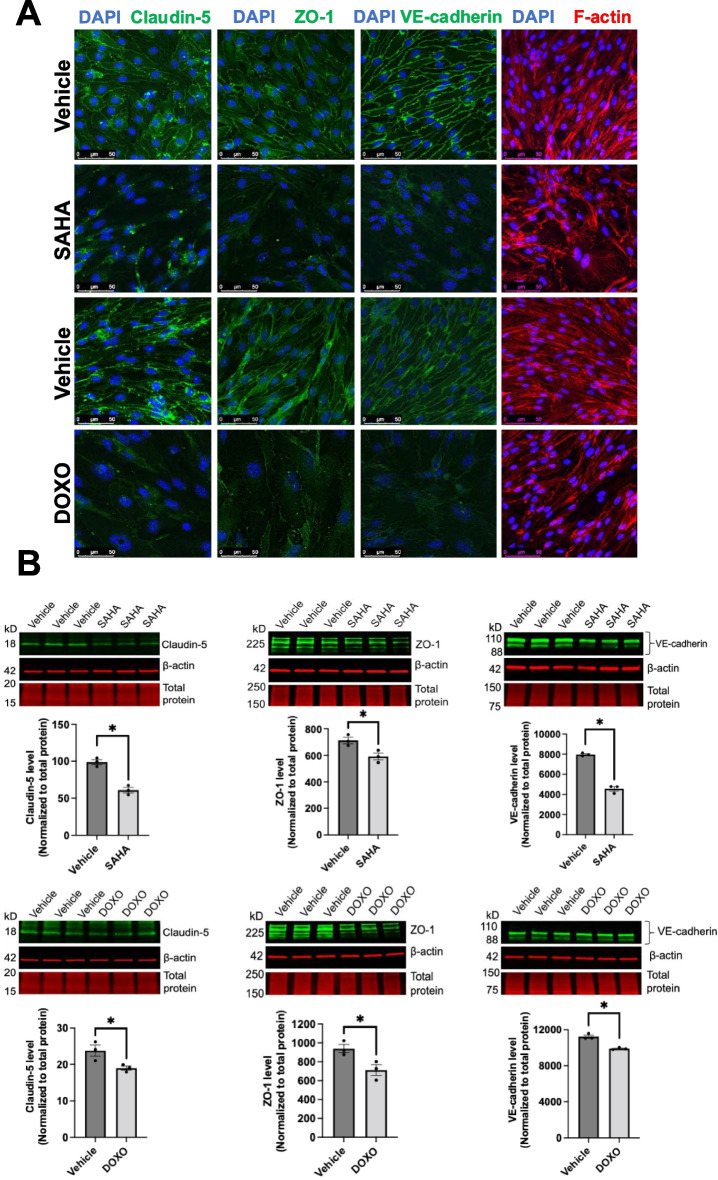
Senescent ECs show decreased expression of cell–cell junction molecules. **A** Representative confocal microscopy demonstrated reduced expression of cell–cell junction barrier molecules (claudin-5, ZO-1, and VE-cadherin) (green) and F-actin (red) in SAHA- or DOXO-treated ECs compared to vehicle group. Nucleus was counterstained with DAPI (blue). Scale bar = 50 μm. **B** Western blotting confirmed lower expression of claudin-5, ZO-1, and VE-Cadherin in SAHA- or DOXO-treated ECs. Data are expressed as mean ± SEM and analyzed by paired Student’s *t*-test; *n* = 3 replicates per group; *P < 0.05. Full-length Western blotting images were provided in Supplemental Fig. S5-S6

To determine the mechanisms whereby senescent ECs exhibited increased permeability, we next evaluated the integrity of the cell–cell junctions. Immunostaining results showed that non-senescent cells had well-organized cell monolayers with visible claudin-5, ZO-1, and VE-cadherin expression at the cell borders, indicating well established cell–cell contacts (Fig. 6A); in contrast, senescent cells showed a disrupted immunostaining pattern with a lot of intercellular gaps, suggesting damage to the integrity of cell junctions in the endothelial monolayer. As endothelial permeability is regulated by contractile cytoskeletal rearrangement [24], we also assessed the changes in the cytoskeleton. Immunofluorescence results revealed that SAHA or doxorubicin-treated lung ECs showed F-actin disorganization compared to control (non-senescent) cells (Fig. 6A). Additionally, we showed a reduction in the expression of claudin-5, ZO-1, and VE-cadherin in ECs after senescence induction (Fig. 6B), corroborating the findings by confocal microscopy.

### Senescent ECs promote neutrophil adhesion and chemotactic migration by upregulating ICAM-1 and secreting chemotactic factors

**Fig. 7:**
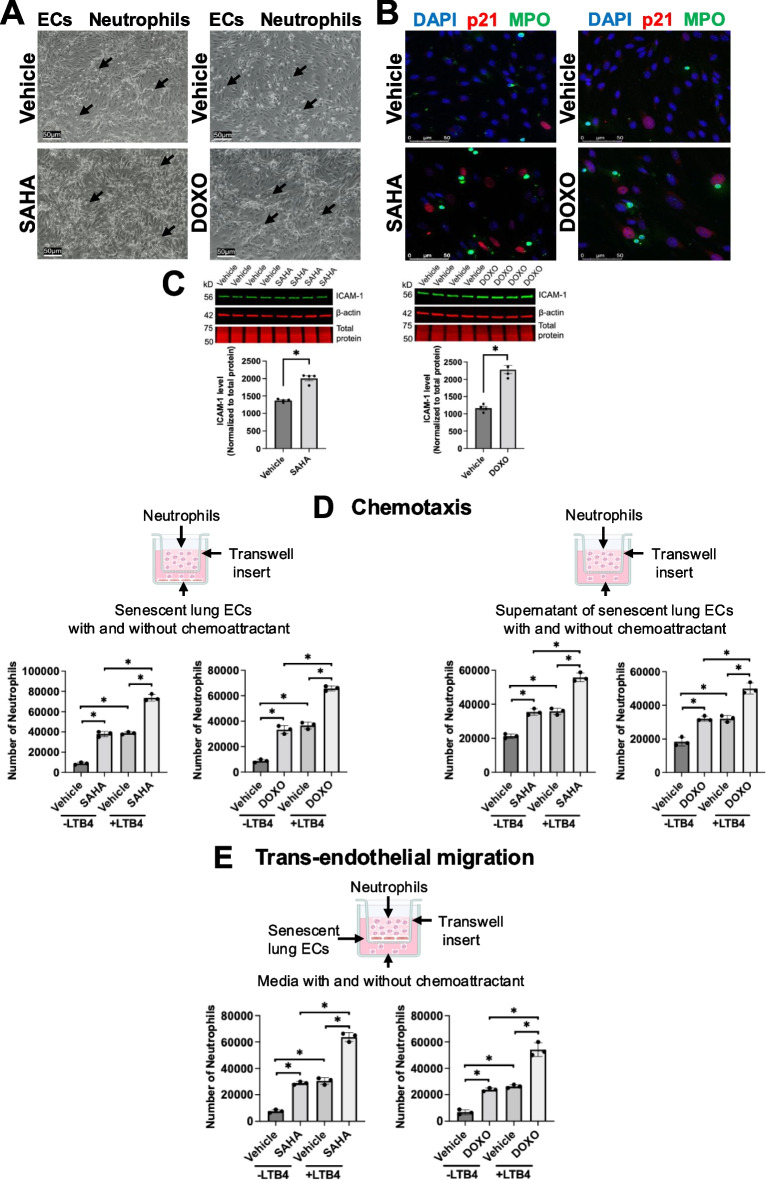
Senescent ECs increase neutrophil adhesion and promote neutrophil chemotaxis and trans-endothelial migration. **A** Representative bright-field microscopy displayed senescent EC monolayer attracted more neutrophils than the non-senescent monolayer. Scale bar = 50 μm.** B** Representative confocal microscopy revealed enhanced adhesion and localization of neutrophils in the vicinity of senescent cells. Nucleus was counterstained with DAPI (blue). Scale bar = 50 μm.** C** Western blotting showed upregulated ICAM-1 in senescent than non-senescent ECs. Data are expressed as mean ± SEM and analyzed by paired Student’s *t*-test; *n* = 4 replicates per group; *P < 0.05. For full-length Western blotting images, see Supplemental Fig. S7. **D** More neutrophils were attracted when senescent ECs or their supernatant were present in the bottom chamber with or without LTB4. Data are expressed as mean ± SEM and analyzed by ANOVA with Tukey’s multiple comparison tests; *n* = 3 replicates per group; *P < 0.05. **E** More neutrophils crossed the senescent EC monolayer than the non-senescent monolayer with or without LTB4 at the bottom chamber. Data are expressed as mean ± SEM and analyzed by ANOVA with Tukey’s multiple comparison tests; *n* = 3 replicates per group; *P < 0.05

To test whether senescent ECs contribute to neutrophil adhesion, we co-cultured senescent mouse primary lung microvascular ECs with mouse bone marrow neutrophils isolated from young mice. Our results showed senescent EC monolayer attracted more neutrophils than the non-senescent monolayer (Fig. 7A). Also, our results revealed enhanced adhesion and localization of neutrophils in the vicinity of senescent cells (Fig. 7B). Western blotting analysis demonstrated increased expression of ICAM-1 in senescent ECs compared to non-senescent cells (Fig. 7C), supporting the finding that senescent ECs enhanced neutrophil recruitment.

To investigate whether senescent ECs affect their interactions with neutrophils, neutrophil chemotaxis and trans-endothelial migration assays were performed. Neutrophil chemotaxis assay showed that more neutrophils were attracted when senescent ECs were present in the bottom chamber with or without a potent neutrophil chemoattractant leukotriene B4 (LTB4) (Fig. 7D), indicating that EC senescence augments neutrophil chemotaxis. In addition, the supernatant from senescent ECs also induced neutrophil chemotaxis (Fig. 7D). Similarly, neutrophil trans-endothelial migration assay demonstrated that more neutrophils crossed the senescent EC monolayer than the non-senescent monolayer with or without LTB4 at the bottom chamber (Fig. 7E).

## Discussion

Cellular senescence is a hallmark of aging that plays a critical role in age-related organ dysfunction and chronic disease states [26]. In this study, we focused on how aging affects EC senescence and barrier integrity in human and mouse lungs. In addition, we evaluated how senescent ECs regulate barrier function and neutrophil infiltration. Our data show that aged human and mouse lungs display increased senescent marker expression and disrupted EC junctions. In vitro, EC senescence impairs junction barrier integrity, increases solute permeability, and facilitates neutrophil adhesion and migration. Our study adds a piece to the puzzle of vascular aging by revealing the impact of senescent ECs on endothelial barrier function and neutrophil-endothelium interactions.

Both aged human and mouse lungs display increased expression of biomarkers for cellular senescence. This is consistent with previous publications demonstrating that the number of senescent cells is increased with age [27]. Since virtually all cells can undergo senescence [28], the senescent characteristics we observed in aged human and mouse lungs could be attributed to different cell types. Given that ECs account for 30% of lung cells [29] and are more susceptible to senescence than other cell types such as epithelial cells [30], senescent ECs may play a dominant role in lung aging.

Aging is a major risk factor for microvascular dysfunction and endothelial barrier leakage. Endothelial cell–cell junctions play an essential role in maintaining barrier integrity and regulating microvascular permeability [16]. A previous study in human umbilical vein ECs shows that senescence induction results in reduced expression of occludin and claudin-5 and affects VE-cadherin and ZO-1 distribution with minor impact on their expression [31]. In contrast, our study revealed that senescent mouse lung microvascular ECs show lower levels of both tight junction and adherens junction proteins, including claudin-5, ZO-1, and VE-cadherin. This discrepancy might be explained by the different nature of senescence induction (repetitive replication vs. chemical-induced), or due to the different cell types (human umbilical vein vs. mouse lung microvascular ECs). Functionally, both senescent human umbilical vein and mouse lung microvascular ECs show increased endothelial permeability. Our study supports that the increased permeability in senescent endothelial monolayers is associated with reduced cell–cell junction protein expression.

“Inflammaging” is coined to describe the inflammatory state of cells and tissues in association with aging and senescence [32]. Senescent cells generally exhibit a SASP phenotype that secrete an array of pro-inflammatory mediators, including IL-1β, IL-6, IL-8, and TNF-α [26]. Older adults display an elevation in circulating and tissue pro-inflammatory cytokines, including IL-1β, IL-6, IL-8, and TNF-α [28]. Alveolar fluid in aged mice and elderly humans also contains enhanced concentration of TNF, IL-6, and complement components [33]. Both IL-1β and TNF-α can directly induce EC barrier dysfunction coupled with TJ and AJ diffusion and disorganization [34]. Therefore, whether the SASP factors partially mediate the impact of senescent ECs on permeability needs further investigation. Our data showed that both senescent ECs and their supernatant promote neutrophil adhesion, chemotaxis, and trans-endothelial migration. Senescent ECs express upregulated ICAM-1 that mediate neutrophil-EC interaction. Therefore, it is likely that senescent ECs facilitate neutrophil infiltration by upregulating ICAM-1 expression and secreting chemotactic factors.

The precise cell sources of SASP in aged lungs remains unclear. Lung macrophages from aged mice generate higher levels of pro-inflammatory mediators, including CCL2, TNF-α, and macrophage migration inhibitory factor (MIF) [35], indicating that macrophages also contribute to inflammation in aged lungs. Neutrophils are also increased in the lungs in healthy, clinically normal, elderly adults [36]. Similarly, our data show that neutrophil numbers are significantly increased in aged mouse lungs. Given the fact that neutrophils can secrete a variety of pro-inflammatory factors [37], neutrophils may also facilitate inflammation in the aged lung. In addition, senescent cells exhibit a distinct SASP phenotype and secrete pro-inflammatory mediators. Thus, senescent ECs may play a role in lung inflammation with age as well.

We acknowledge a few limitations in our study. First, our study focused on how lung EC senescence affects lung aging. Whether and how other cell types, such as epithelial cells and alveolar macrophages, contribute to inflammaging in the lungs or other organs can be evaluated in separate studies. Second, the molecular details or signaling mechanisms underlying senescence-induced endothelial barrier dysfunction require further investigation. For example, cytosolic phospholipase A_2_ (cPLA_2_α) regulates EC junction integrity [38], and its expression is significantly lower in senescent ECs [31]. Whether overexpressing cPLA_2_α could restore barrier function in senescent ECs is an open question. Third, neutrophils from old adults demonstrate functional impairment, including impaired formation of neutrophil extracellular traps (NETs), reduced chemotaxis, and inaccurate migration [39, 40]. In our study, we used neutrophils isolated from young mice. It is warranted to unravel how senescent ECs regulate the activation and functions of aged neutrophils. Fourth, we used the transwell system to evaluate neutrophil chemotaxis and trans-endothelial migration. It is possible that some neutrophils may stick to the membrane, and the neutrophil number in the bottom chamber is underestimated.

In summary, our study provides novel evidence supporting the crucial role of senescent ECs in promoting barrier dysfunction and neutrophil recruitment during aging. This increases our understanding of the molecular pathogenesis of aging-related pathologies in the lungs.

## Supplementary Information

Below is the link to the electronic supplementary material.

Supplementary file1 (PDF 145 KB)

Supplementary file2 (PDF 138 KB)

Supplementary file3 (PDF 105 KB)

Supplementary file4 (PDF 231 KB)

Supplementary file5 (PDF 177 KB)

Supplementary file6 (PDF 187 KB)

Supplementary file7 (PDF 136 KB)

Supplementary file8 (DOCX 17 KB)

## Data Availability

Data will be available upon request.

## Abbreviations

AJs: Adherens junctions
DOXO: Doxorubicin
ECs: Endothelial cells
ICAM-1: Intercellular adhesion molecule 1
MPO: Myeloperoxidase
PFA: Paraformaldehyde
SA-β-Gal: Senescence-associated-β-galactosidase
SASP: Senescence-associated secretory phenotype
TJs: Tight junctions
