# Supplementary figures and images for: Lung endothelial cell senescence impairs barrier function and promotes neutrophil adhesion and migration
Source: GeroScience. 2025 Jan 16;47(3):2655–71. doi: 10.1007/s11357-025-01517-9 (PMC12181458; doi:10.1007/s11357-025-01517-9)

### p21

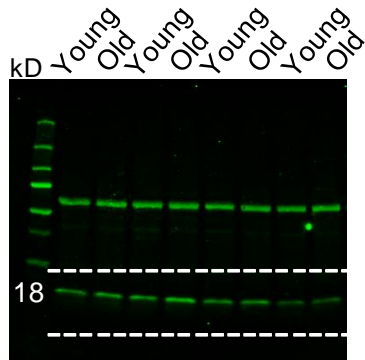

### Lamin B1

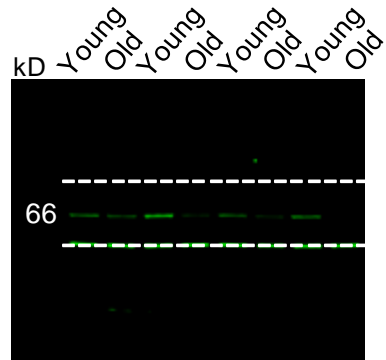

### $\beta$ -actin

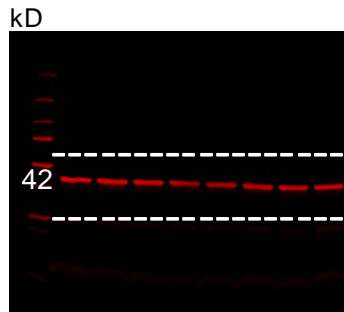

### $\beta$ -actin

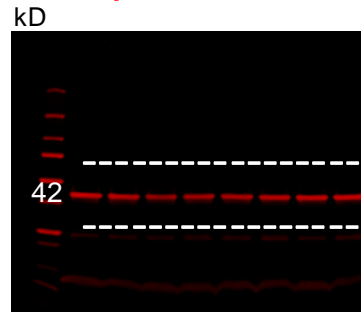

### Total protein

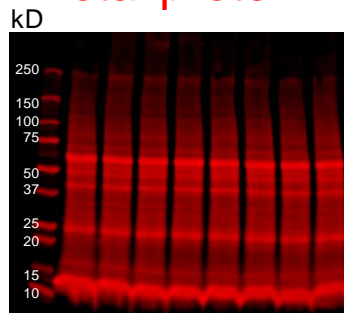

### Total protein

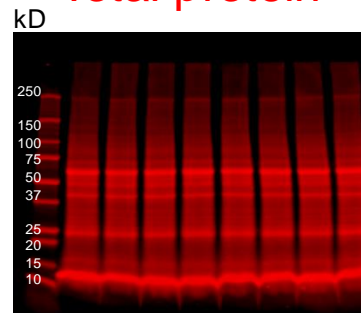

Supplement: Supplementary file 1 — Supplementary file1 (PDF 145 KB) [file 11357_2025_1517_MOESM1_ESM.pdf]

## ZO-1

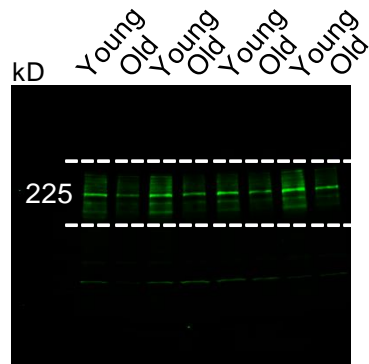

## VE-cadherin

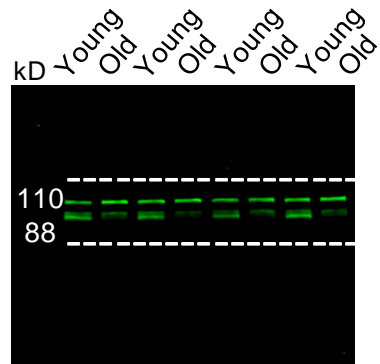

## $\beta$ -actin

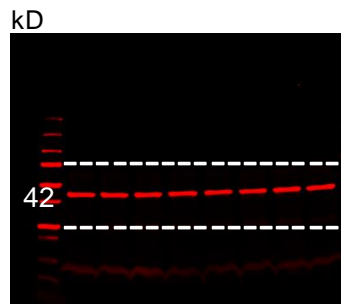

## $\beta$ -actin

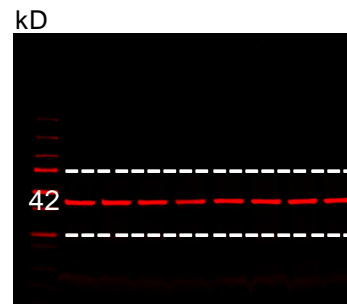

## Total protein

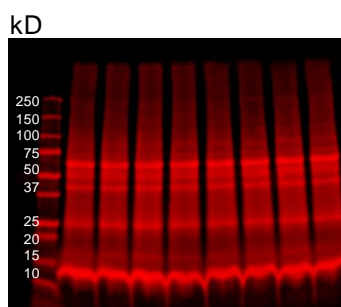

## Total protein

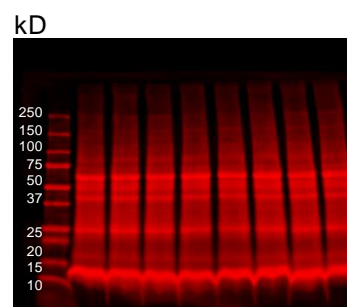

Supplement: Supplementary file 2 — Supplementary file2 (PDF 138 KB) [file 11357_2025_1517_MOESM2_ESM.pdf]

ICAM-1

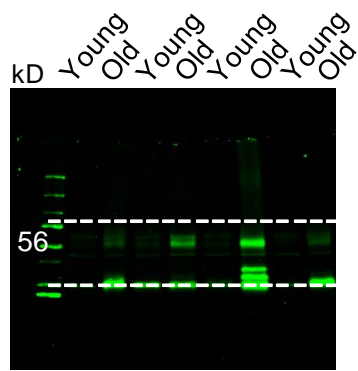

$\beta$ -actin

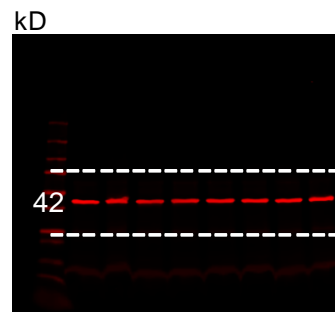

Total protein

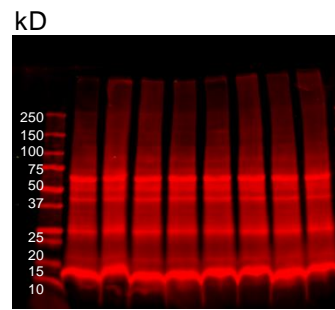

Supplement: Supplementary file 3 — Supplementary file3 (PDF 105 KB) [file 11357_2025_1517_MOESM3_ESM.pdf]

ICAM-1

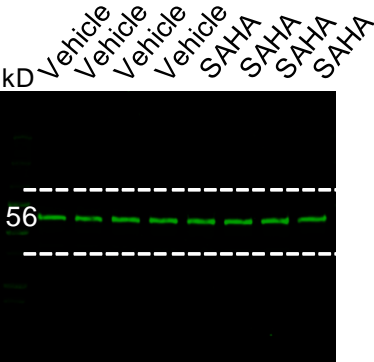

ICAM-1

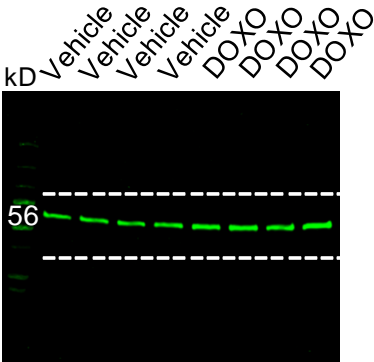

$\beta$ -actin

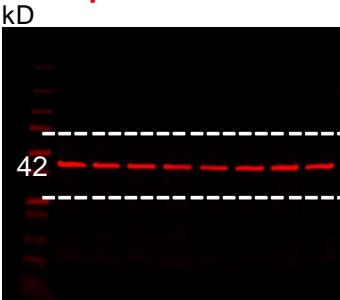

$\beta$ -actin

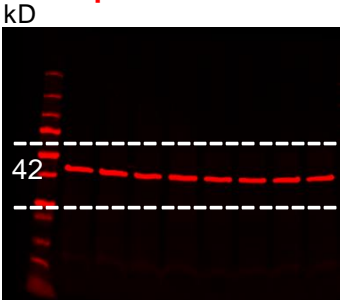

Total protein

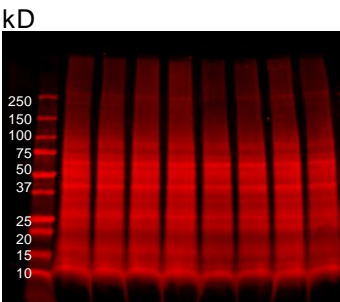

Total protein

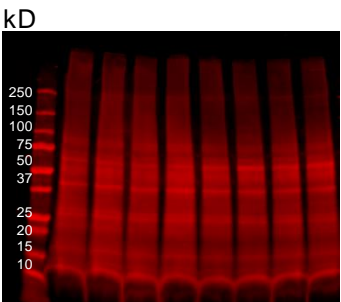

Supplement: Supplementary file 7 — Supplementary file7 (PDF 136 KB) [file 11357_2025_1517_MOESM7_ESM.pdf]
