# Supplementary material for: Lung endothelial cell senescence impairs barrier function and promotes neutrophil adhesion and migration
Source: GeroScience. 2025 Jan 16;47(3):2655–71. doi: 10.1007/s11357-025-01517-9 (PMC12181458; doi:10.1007/s11357-025-01517-9)
Supplement: Supplementary file 4 — Supplementary file4 (PDF 231 KB) [file 11357_2025_1517_MOESM4_ESM.pdf]

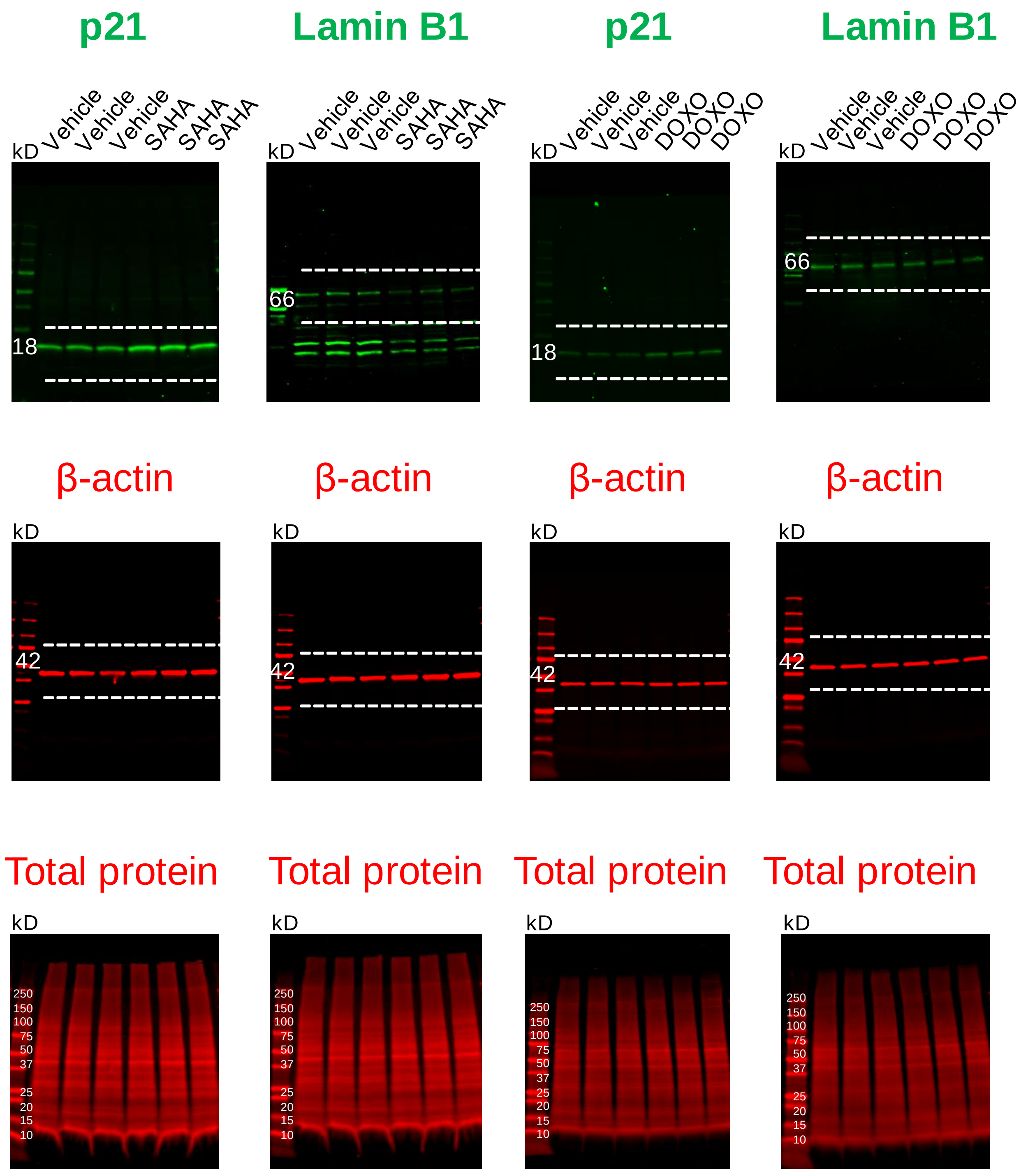

p21
Lamin B1
p21
Lamin B1
kD
Vehicle
Vehicle
Vehicle
SAHA
SAHA
SAHA
18
kD
Vehicle
Vehicle
Vehicle
SAHA
SAHA
SAHA
66
kD
Vehicle
Vehicle
Vehicle
DOXO
DOXO
DOXO
18
kD
Vehicle
Vehicle
Vehicle
DOXO
DOXO
DOXO
66
β-actin
β-actin
β-actin
β-actin
kD
42
kD
42
kD
42
kD
42
Total protein
Total protein
Total protein
Total protein
kD
250
150
100
75
50
37
25
20
15
10
kD
250
150
100
75
50
37
25
20
15
10
kD
250
150
100
75
50
37
25
20
15
10
kD
250
150
100
75
50
37
25
20
15
10
