# Supplementary material for: Lung endothelial cell senescence impairs barrier function and promotes neutrophil adhesion and migration
Source: GeroScience. 2025 Jan 16;47(3):2655–71. doi: 10.1007/s11357-025-01517-9 (PMC12181458; doi:10.1007/s11357-025-01517-9)
Supplement: Supplementary file 6 — Supplementary file6 (PDF 187 KB) [file 11357_2025_1517_MOESM6_ESM.pdf]

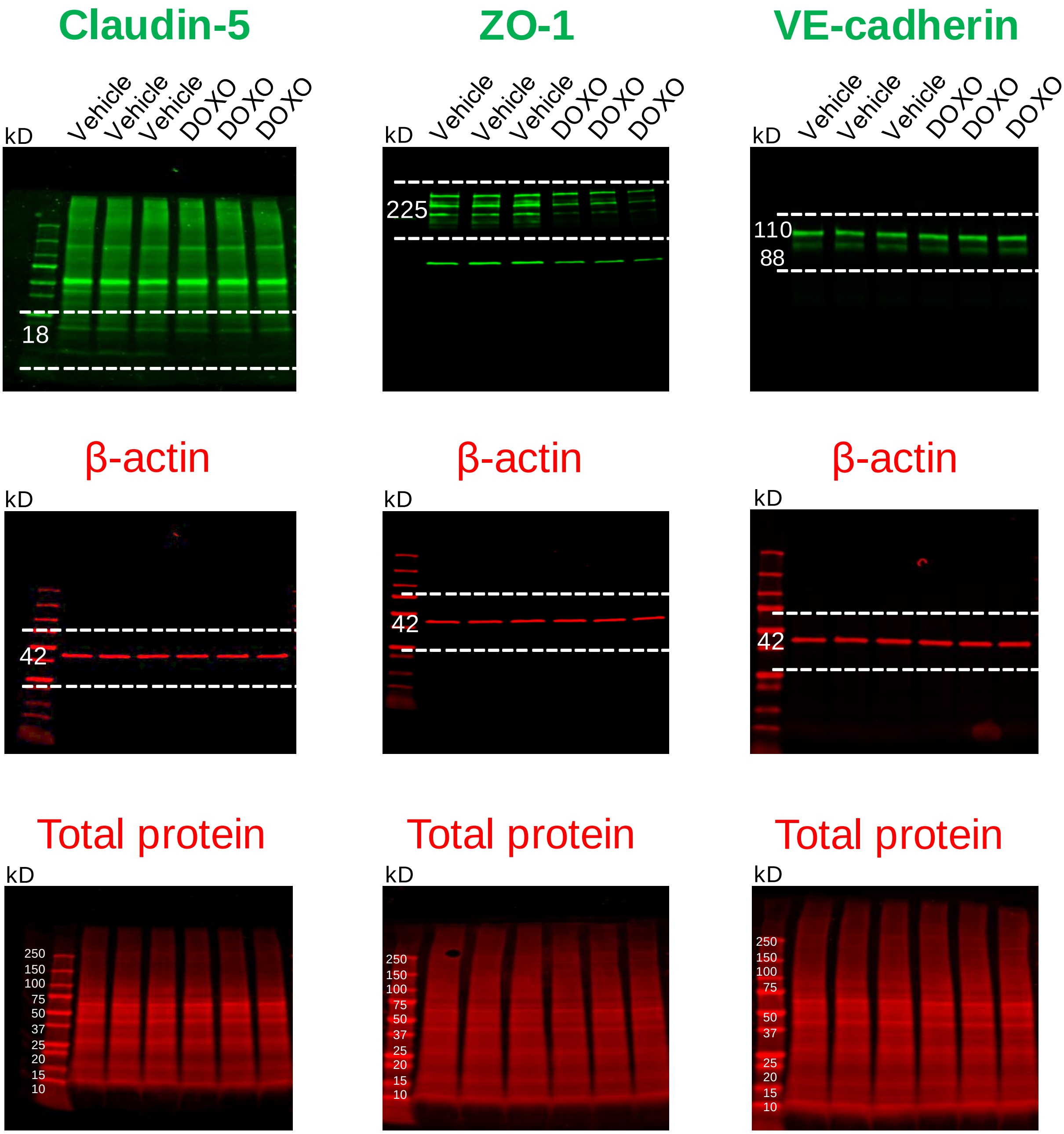

Claudin-5
ZO-1
VE-cadherin
Vehicle
Vehicle
Vehicle
DOXO
DOXO
DOXO
kD
18
225
110
88
β-actin
42
Total protein
250
150
100
75
50
37
25
20
15
10
