# Supplementary material for: Lung endothelial cell senescence impairs barrier function and promotes neutrophil adhesion and migration
Source: GeroScience. 2025 Jan 16;47(3):2655–71. doi: 10.1007/s11357-025-01517-9 (PMC12181458; doi:10.1007/s11357-025-01517-9)
Supplement: Supplementary file 8 — Supplementary file8 (DOCX 17 KB) [file 11357_2025_1517_MOESM8_ESM.docx]

**Supplementary Materials**

**Lung Endothelial Cell Senescence Impairs Barrier Function and Promotes Neutrophil Adhesion and Migration**

Maliheh Najari Beidokhti^1^, Nuria Villalba^1^, Yonggang Ma^1^, Amanda Reynolds^1^, Juan Hernandez Villamil^1^, Sarah Y. Yuan^1,2,^*

^1^Department of Molecular Pharmacology and Physiology, University of South Florida, Morsani College of Medicine, Tampa, FL, USA. ^2^Department of Surgery, University of South Florida, Morsani College of Medicine, Tampa, FL, USA.

*Corresponding author:

Sarah Y. Yuan, M.D., Ph.D.

Department of Molecular Pharmacology and Physiology

University of South Florida, Morsani College of Medicine

12901 Bruce B. Downs Blvd. Tampa, FL, U.S.A.

Phone: +1 (813)-974-0210 / email: [syuan@usf.edu](mailto:syuan@usf.edu)

**ACKNOWLEDGMENTS**

We thank Dr. Byeong Jake Cha for providing technical expertise with confocal microscopy. We also thank Mr. Sam Spence for his assistance with neutrophil isolation experiments. This work was supported by the National Institutes of Health grants HL150732 and GM142110 (to S.Y.Y).

**Figure S1.** **Full-length Western blotting images of p21 and Lamin B1 expression in mouse lungs**

Expression levels of p21 and Lamin B1 markers (top panels) and β-actin (middle panels) in mouse lung tissue. Full-length scans of total protein staining (bottom panels) used for quantification in main figures. Western blots of senescent cell markers p21 and Lamin B1 were normalized to total protein (n=4 mice per group).

**Figure S2.** **Full-length Western blotting images of ZO-1 and VE-cadherin expression in mouse lungs**

Expression levels of ZO-1 and VE-cadherin markers (top panels) and β-actin (middle panels) in mouse lung tissue. Full-length scans of total protein staining (bottom panels) used for quantification in main figures. Western blots of barrier molecules ZO-1 and VE-cadherin were normalized to total protein (n=4 mice per group).

**Figure S3.** **Full-length Western blotting image of ICAM-1 expression in mouse lungs**

Expression level of ICAM-1 marker (top panel) and β-actin (middle panel) in mouse lung tissue. Full-length scan of total protein staining (bottom panel) used for quantification in main figure. Western blot of adhesion molecule ICAM-1 was normalized to total protein (n=4 mice per group).

**Figure S4. Full-length Western blotting images of p21 and Lamin B1 expression in cultured mouse lung microvascular endothelial cells, treated with two senescent inducers, SAHA, and doxorubicin vs vehicle-treated cells**

Expression levels of p21 and Lamin B1 markers (top panels) and β-actin (middle panels) in cultured mouse lung microvascular endothelial cells, treated with two senescent inducers, SAHA, and doxorubicin vs vehicle-treated cells. Full-length scans of total protein staining (bottom panels) used for quantification in main figures. Western blots of senescent cell markers p21 and Lamin B1 were normalized to total protein (n=3 replicates per group).

**Figure S5. Full-length Western blotting images of claudin-5, ZO-1, and VE-cadherin expression in cultured mouse lung microvascular endothelial cells, treated with senescent inducer, SAHA, vs vehicle-treated cells**

Expression levels of claudin-5, ZO-1 and VE-cadherin markers (top panels) and β-actin (middle panels) in cultured mouse lung microvascular endothelial cells, treated with senescent inducer, SAHA, vs vehicle-treated cells. Full-length scans of total protein staining (bottom panels) used for quantification in main figures. Western blots of cell-cell junction molecules claudin-5, ZO-1 and VE-cadherin were normalized to total protein (n=3 replicates per group).

**Figure S6. Full-length Western blotting images of claudin-5, ZO-1, and VE-cadherin expression in cultured mouse lung microvascular endothelial cells, treated with senescent inducer, doxorubicin, vs vehicle-treated cells**

Expression levels of claudin-5, ZO-1 and VE-cadherin markers (top panels) and β-actin (middle panels) in cultured mouse lung microvascular endothelial cells, treated with senescent inducer, doxorubicin, vs vehicle-treated cells. Full-length scans of total protein staining (bottom panels) used for quantification in main figures.

Western blots of cell-cell junction molecules claudin-5, ZO-1 and VE-cadherin were normalized to total protein (n=3 replicates per group).

**Figure S7. Full-length Western blotting images of ICAM-1 expression in cultured mouse lung microvascular endothelial cells, treated with two senescent inducers, SAHA, and doxorubicin vs vehicle-treated cells**

Expression level of ICAM-1 marker (top panel) and β-actin (middle panel) in cultured mouse lung microvascular endothelial cells, treated with two senescent inducers, SAHA, and doxorubicin vs vehicle-treated cells. Full-length scans of total protein staining (bottom panel) used for quantification in main figures. Western blots of adhesion molecule ICAM-1 were normalized to total protein (n=4 replicates per group).
